# Supplementary material for: Extracellular DNA released by glycine-auxotrophic Staphylococcus epidermidis small colony variant facilitates catheter-related infections
Source: Commun Biol. 2021 Jul 22;4:904. doi: 10.1038/s42003-021-02423-4 (PMC8298460; doi:10.1038/s42003-021-02423-4)
Supplement: Supplementary file 1 — Supplementary Information [file 42003_2021_2423_MOESM1_ESM.pdf]

Extracellular DNA released by glycine-auxotrophic *Staphylococcus epidermidis* small colony variant facilitates catheter-related infections

Junlan Liu[1], Zhen Shen[1], Jin Tang[2], Qian Huang[1], Ying Jian[1], Yao Liu[1], Yanan Wang[1], Xiaowei Ma[1], Qian Liu[1], Lei He*[1], Min Li*[1,3]

1: Department of Laboratory Medicine, Renji Hospital, School of Medicine, Shanghai Jiaotong University, Shanghai 200127, China

2: Department of Laboratory Medicine, Shanghai Jiaotong University Affiliated Sixth People's Hospital, Shanghai 200233, China

3: Faculty of medical laboratory science, Shanghai Jiao tong University School of Medicine, Shanghai 200025, China.

*Correspondence:

Corresponding Authors email:

Lei He : buningweishi_1985@126.com

Min Li : rjlimin@shsmu.edu.cn

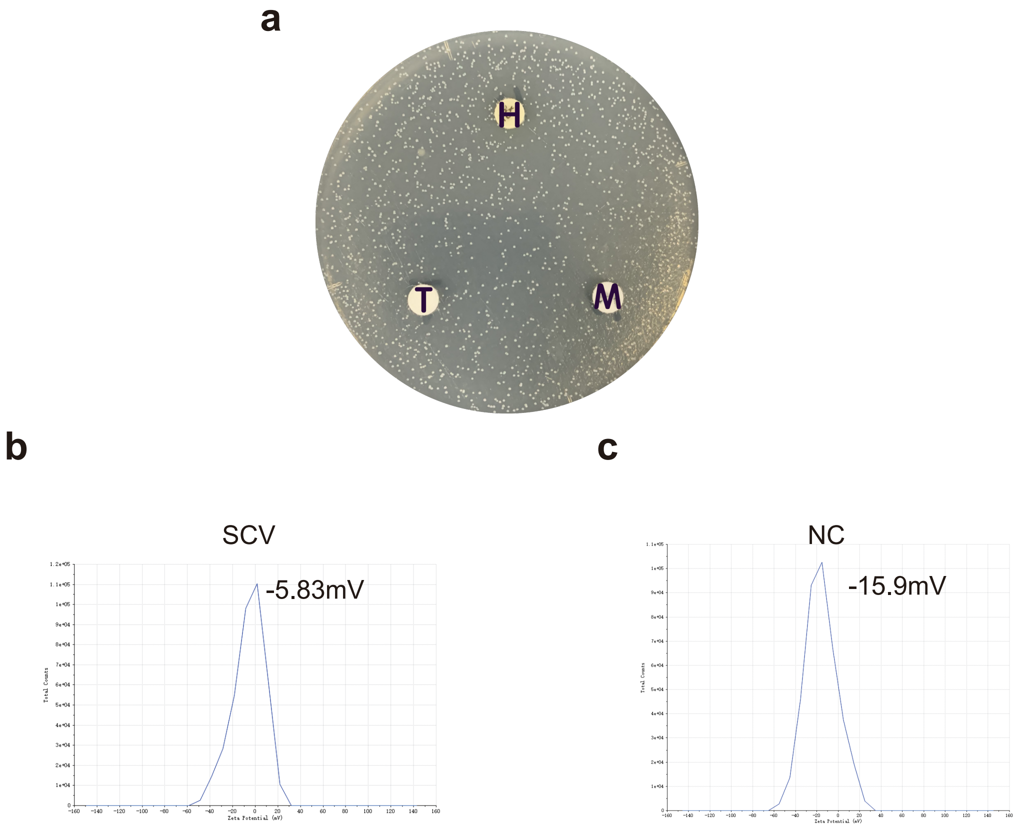

Supplementary Figure 1. a No auxotrophy of either hemin or menadione, or thymidine (T) was demonstrated by the *S. epidermidis* stable SCV isolate. Paper disks impregnated with 1.5 μg of hemin (H), menadione (M) and thymidine (T) were placed onto a Mueller–Hinton agar plate spread with bacterial culture in advance. The plate was then incubated at 37 °C for 24 hours. **b** Zeta potential distribution of SCV. **c** Zeta potential distribution of NC. Mean values of zeta potential were indicated.

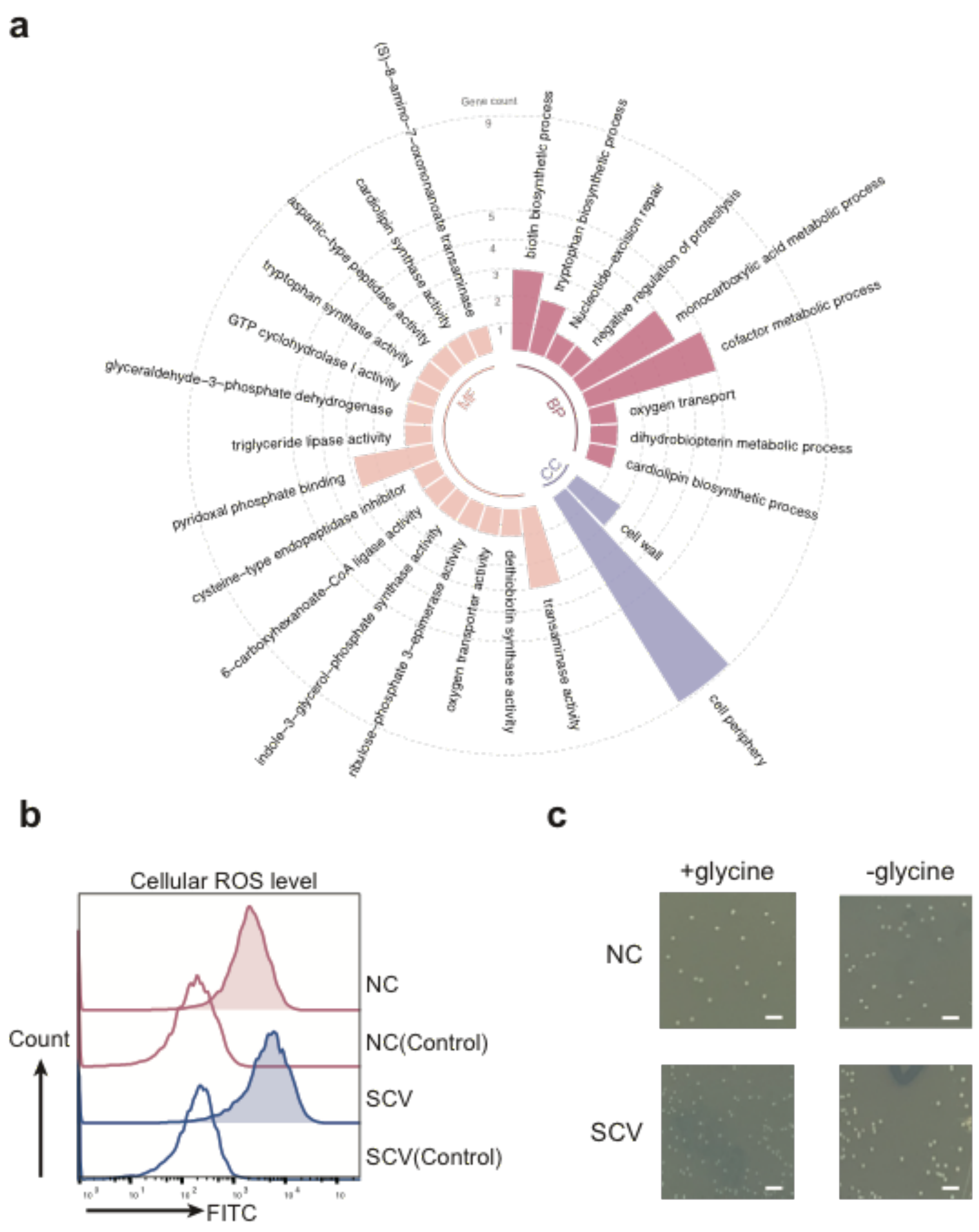

Supplementary Figure 2. a Enriched gene ontology (GO) terms (enrichment P value <0.05) for the mutation-related gene set. GO Terms were classified as biological process (BP), cellular component (CC) and molecular function (MF). Height of the bar represents the absolute number of genes included in the indicated GO term. **b** Intracellular ROS level. **c** *S. epidermidis* stable SCV and its NC grown on agar plates after 24 hours of incubation in either the absence or the presence of glycine. The bottom bars equal 2 mm.

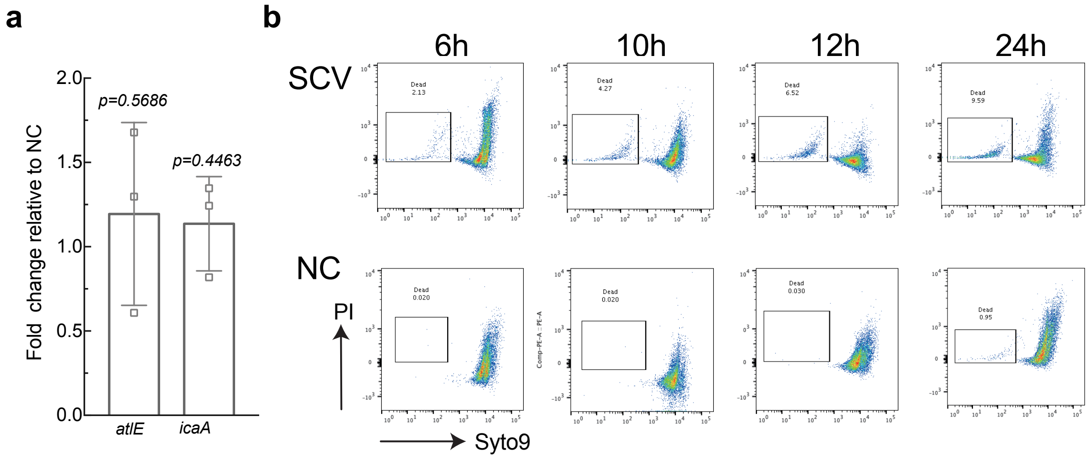

Supplementary Figure 3. a No significant transcriptional differences of *icaA* and *atlE* were observed in *S. epidermidis* stable SCV. Data shown are mean ± sd from three biological replicates. **b** Viability analysis of batch cultures using PI and Syto9. The Percentages of Dead population were indicated.

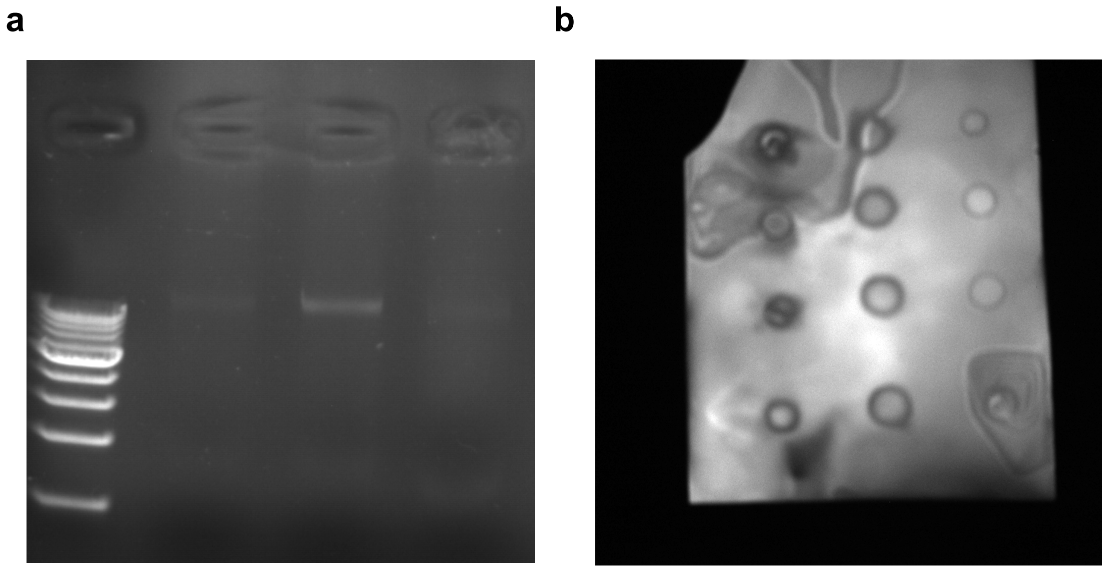

Supplementary Figure 3. a The raw agarose gel image. **b** The raw blot image.

Supplementary Table 1. Isolates included in this study

Isolate	abbreviation	Source of isolation	Propertiesa	Susceptibility of antimicrobial agents									
				AMK	ERY	CZO	TET	SXT	RIF	LEV	DA	LZD	VAN
xh55-SCV	Stable SCV	Intravenous catheter from a two-month-old patient	Stable SCV, MRSE-ST2-agr I	R	R	R	R	R	R	I	R	S	S
xh55-NC	NC		Normal-sized MRSE-ST2-agr I	R	R	R	R	R	R	I	R	S	S
xh55-Rev	Rev		Revertant of instable SCV, MRSE-ST2-agr I	R	R	R	R	R	R	I	R	S	S

a, MRSE, methicillin-resistant *Staphylococcus epidermidis*; MSSE, methicillin-sensitive *Staphylococcus epidermidis*; ST: sequence type.

b, abbreviations: AMK, amikacin; ERY, erythromycin; CZO, cefazolin; TET, tetracycline; SXT, trimethoprim-sulfamethoxazole; RIF, rifampin; LEV, levofloxacin; DA, clindamycin; LZD, linezolid; VAN, vancomycin;

Supplementary Table 2. Primers used in qRT-PCR assays.

Gene	Direction	Sequences (5'→3')
gyrB	Forward	AAGGGTATTATGGCTTCACG
	Reverse	TTTCACTTTCTTCAGGGTTC
atlE	Forward	AACGAAGCAAGTAGCACC
	Reverse	ACACCACGATTAGCAGAC
icaA	Forward	GCACTCAATGAGGGAATCA
	Reverse	TAACTGCGCCTAATTTTGGATT
cidA	Forward	GCAGGTAGTATCGTAGGG
	Reverse	CCTACCACTGATGGGATA
lrgA	Forward	CTGTGGGAACTGCATTAACC
	Reverse	GTGACGCAAAGCCAGTACAA
