# Supplementary material for: Extracellular DNA released by glycine-auxotrophic Staphylococcus epidermidis small colony variant facilitates catheter-related infections
Source: Commun Biol. 2021 Jul 22;4:904. doi: 10.1038/s42003-021-02423-4 (PMC8298460; doi:10.1038/s42003-021-02423-4)
Supplement: Supplementary file 4 — Reporting Summary [file 42003_2021_2423_MOESM4_ESM.pdf]

nature research

Corresponding author(s): Min Li and Lei He

Last updated by author(s): Jun 13, 2021

Reporting Summary

Nature Research wishes to improve the reproducibility of the work that we publish. This form provides structure for consistency and transparency in reporting. For further information on Nature Research policies, see our Editorial Policies and the Editorial Policy Checklist.

Statistics

For all statistical analyses, confirm that the following items are present in the figure legend, table legend, main text, or Methods section.

n/a	Confirmed	
☐	☒	The exact sample size (*n*) for each experimental group/condition, given as a discrete number and unit of measurement
☐	☒	A statement on whether measurements were taken from distinct samples or whether the same sample was measured repeatedly
☐	☒	The statistical test(s) used AND whether they are one- or two-sided<br>*Only common tests should be described solely by name; describe more complex techniques in the Methods section.*
☒	☐	A description of all covariates tested
☐	☒	A description of any assumptions or corrections, such as tests of normality and adjustment for multiple comparisons
☐	☒	A full description of the statistical parameters including central tendency (e.g. means) or other basic estimates (e.g. regression coefficient) AND variation (e.g. standard deviation) or associated estimates of uncertainty (e.g. confidence intervals)
☒	☐	For null hypothesis testing, the test statistic (e.g. F, t, r) with confidence intervals, effect sizes, degrees of freedom and P value noted<br>*Give P values as exact values whenever suitable.*
☒	☐	For Bayesian analysis, information on the choice of priors and Markov chain Monte Carlo settings
☒	☐	For hierarchical and complex designs, identification of the appropriate level for tests and full reporting of outcomes
☒	☐	Estimates of effect sizes (e.g. Cohen's d, Pearson's r), indicating how they were calculated

Our web collection on statistics for biologists contains articles on many of the points above.

Software and code

Policy information about availability of computer code

Data collection	As described in the "Materials and Methods" section. Data was collected either manually or by BD FACSuite software, ZEISS ZEN Blue, and BioTek's Gen5 and Synergy H1 Software.
Data analysis	As described in the "Materials and Methods" section. Data analyses were performed using Prism 7.0a, FlowJo 10.4, MetaboAnalyst (http://www.metaboanalyst.ca), SIMCA15.0.2, ZEISS ZEN Black , COMSTAT2 and ImageJ 1.53a.

For manuscripts utilizing custom algorithms or software that are central to the research but not yet described in published literature, software must be made available to editors and reviewers. We strongly encourage code deposition in a community repository (e.g. GitHub). See the Nature Research guidelines for submitting code & software for further information.

Data

Policy information about availability of data

All manuscripts must include a data availability statement. This statement should provide the following information, where applicable:

- Accession codes, unique identifiers, or web links for publicly available datasets
- A list of figures that have associated raw data
- A description of any restrictions on data availability

Sequencing data available in the SRA database under the accession number: PRJNA714433. All data underlying the main and Supplementary figures are either available online, or available from the corresponding authors [LM and HL], upon reasonable request.

Field-specific reporting

Please select the one below that is the best fit for your research. If you are not sure, read the appropriate sections before making your selection.

- [x] Life sciences
- [ ] Behavioural & social sciences
- [ ] Ecological, evolutionary & environmental sciences

For a reference copy of the document with all sections, see nature.com/documents/nr-reporting-summary-flat.pdf

Life sciences study design

All studies must disclose on these points even when the disclosure is negative.

Sample size	The sample size of catheter-infection cases was determined primarily based on the culture-positive rate of catheter specimens, cost and time for collecting data.
Data exclusions	Data were excluded from analysis when they were abnormally different from other replicates.
Replication	Biological and technical replicates were included and indicated in the manuscript.
Randomization	Mice were selected randomly for experiments and randomization is not needed for other parts of experiments.
Blinding	Blinding was using during CFU counting.

Reporting for specific materials, systems and methods

We require information from authors about some types of materials, experimental systems and methods used in many studies. Here, indicate whether each material, system or method listed is relevant to your study. If you are not sure if a list item applies to your research, read the appropriate section before selecting a response.

Materials & experimental systems

n/a	Involved in the study	
x		Antibodies
x		Eukaryotic cell lines
x		Palaeontology and archaeology
	x	Animals and other organisms
x		Human research participants
x		Clinical data
x		Dual use research of concern

Methods

n/a	Involved in the study	
x		ChIP-seq
	x	Flow cytometry
x		MRI-based neuroimaging

Animals and other organisms

Policy information about studies involving animals; ARRIVE guidelines recommended for reporting animal research

Laboratory animals	Female BALB/c mice (4- to 6-week) of SPF grade were purchased from Shanghai JSJ Laboratory Animal Co, Ltd.
Wild animals	None of wild animals was invovled in this study.
Field-collected samples	None of field-collected samples was invovled in this study.
Ethics oversight	All animal work was approved by the ethics committee of Renji Hospital, School of Medicine, Shanghai Jiao Tong University, Shanghai, China.

Note that full information on the approval of the study protocol must also be provided in the manuscript.

Flow Cytometry

Plots

Confirm that:

☒ The axis labels state the marker and fluorochrome used (e.g. CD4-FITC).

☒ The axis scales are clearly visible. Include numbers along axes only for bottom left plot of group (a 'group' is an analysis of identical markers).

☒ All plots are contour plots with outliers or pseudocolor plots.

☒ A numerical value for number of cells or percentage (with statistics) is provided.

Methodology

Sample preparation	Bacteria were washed twice with PBS after being labeled for half an hour with TAMRA-bearing FDAA probe, or an hour for Syto9 and PI.
Instrument	BD FACSverse system.
Software	BD FACSuite software.
Cell population abundance	No post-sorting was invovled in this study.
Gating strategy	SSC gate was used to identify bacterial cells. Single-staining samples were used for compensation and identifying positive population in double-staining samples.

☒ Tick this box to confirm that a figure exemplifying the gating strategy is provided in the Supplementary Information.
