# Supplementary material for: Extracellular DNA released by glycine-auxotrophic Staphylococcus epidermidis small colony variant facilitates catheter-related infections
Source: Commun Biol. 2021 Jul 22;4:904. doi: 10.1038/s42003-021-02423-4 (PMC8298460; doi:10.1038/s42003-021-02423-4)
Supplement: Supplementary file 5 — Description of Supplementary Files [file 42003_2021_2423_MOESM5_ESM.pdf]

Description of Additional Supplementary Files

File name: Supplementary data 1

Description: Details of Indels and nonsynonymous SNVs specifically found in S. epidermidis SCV genome.

File name: Supplementary data 2

Description: Source data for graphs and charts.
